# The role of testosterone in chronic kidney disease and kidney function in men and women: a bi-directional Mendelian randomization study in the UK Biobank

**DOI:** 10.1186/s12916-020-01594-x

**Published:** 2020-06-04

**Authors:** Jie V. Zhao, C. Mary Schooling

**Affiliations:** 1 School of Public Health, Li Ka Shing Faculty of Medicine, The University of Hong Kong, 1/F, Patrick Manson Building, 7 Sassoon Road, Pokfulam, Hong Kong SAR, China; 2 School of Public Health and Health Policy, City University of New York, New York, NY USA

**Keywords:** Big data, Testosterone, Chronic disease, Mendelian randomization

## Abstract

**Background:**

Chronic kidney disease (CKD) has an apparent sex disparity, with a more rapid progress in men than in women. Whether the well-established sex-specific evolutionary biology trade-off between reproduction and longevity might inform CKD has not previously been considered. Relevant evidence from randomized controlled trials (RCTs) is not available.

**Methods:**

We used a bi-directional Mendelian randomization study to obtain unconfounded estimates using the UK Biobank. Single nucleotide polymorphisms (SNPs) that strongly (*p* value < 5 × 10^−8^) predicted testosterone in a sex-specific manner were applied to 179,916 white British men (6016 CKD cases) and 212,079 white British women (5958 CKD cases) to obtain sex-specific associations with CKD, albuminuria, and estimated glomerular filtration rate (eGFR). We also used multivariable MR to control for sex hormone binding globulin (SHBG). For validation, we similarly examined their role in hemoglobin and high-density lipoprotein cholesterol (HDL-c). We also assessed the role of kidney function in serum testosterone, by applying eGFR-related SNPs to testosterone in the UK Biobank.

**Results:**

Genetically predicted testosterone was associated with CKD in men (odds ratio (OR) for bioavailable testosterone 1.17 per standard deviation, 95% confidence interval (CI) 1.03 to 1.33) based on 125 SNPs but not in women (OR 1.02, 95% CI 0.92 to 1.14 for total testosterone) based on 254 SNPs. Multivariable MR allowing for SHBG showed consistent patterns. Genetically predicted bioavailable testosterone in men and women and genetically predicted total testosterone in women increased hemoglobin and lowered HDL-c, as seen in RCTs. Genetically predicted eGFR was not related to serum testosterone in men or in women.

**Conclusions:**

Genetically predicted testosterone was associated with CKD and worse kidney function in men, whilst not affected by kidney function. Identifying drivers of testosterone and the underlying pathways could provide new insights into CKD prevention and treatment.

## Background

Chronic kidney disease (CKD) represents a major burden of morbidity and mortality globally, affecting 10% of the population worldwide [1]. Renal replacement therapy (dialysis or kidney transplantation), which is the treatment to enable survival during end-stage renal disease, has become the leading cost in health care systems in developed countries [2]. Notably, CKD has an apparent disparity in men and women [3]. Specifically, kidney function declines faster in men than in women, and the mortality rate is higher in men at all levels of pre-dialysis CKD [3]. Understanding the reasons underlying men’s disadvantage may provide new clues about etiology, with relevance to identification of new modifiable targets in primary care and development of clinical treatment for CKD. It may also provide information that can be used to address the sexual disparity in life expectancy.

Accumulating evidence suggests androgens may play a key role in differences in longevity by sex [4]. From the perspective of evolutionary biology, growth and reproduction trade off against longevity, with men and women having different investments in these trade-offs and as a result have different vulnerabilities to disease [4–6]. Correspondingly, hypothalamic-pituitary axis dysfunction is closely related to infertility and to CKD [7, 8], supporting a role of reproductive factors in CKD. Animal experiments have repeatedly shown that testosterone damages renal function [9, 10]. For example, in vitro experiments show testosterone induced renal tubular epithelial cell death in a dose-response manner [10]. Correspondingly, castration in males increases renal clearance [11], lowers proteinuria, and protects against glomerular injury assessed from renal morphology [11, 12]. Nevertheless, evidence, especially experimental evidence, in humans is limited. A clinical case report showed testosterone therapy induced renal impairment [13]. A randomized controlled trial in 48 men showed 6-month testosterone treatment lowered estimated glomerular filtration rate (eGFR) [14]. In contrast, some observational studies have found higher testosterone associated with lower mortality in CKD patients [15] and higher eGFR [16]. These observed associations in patients are difficult to interpret because observational studies, essentially of survivors, are open to selection bias [17]. No large-scale RCT has ever been implemented to assess the long-term effect of testosterone on CKD. Moreover, no study has specifically examined the sex-specific role of testosterone in CKD and kidney function.

In this situation where a large RCT is not available, using naturally occurring testosterone-related genetic variants as proxies of exposure, i.e., a Mendelian randomization (MR) study design, enables us to obtain unconfounded associations without any potentially harmful interventions. Genetic variants resulting in lifelong differences in endogenous exposures are unlikely to be associated with socioeconomic position or other confounders as they are determined at conception [18]. In this study, for the first time, we examined the association of genetically predicted testosterone with CKD events and kidney function using an MR study in the UK Biobank. For validation of the genetic instrument, we similarly examined the effect of genetically predicted testosterone on hemoglobin and high-density lipoprotein (HDL)-cholesterol, because meta-analysis of RCTs has clearly shown testosterone increases hemoglobin and lowers HDL-cholesterol [19]. As serum testosterone may also be affected by kidney function [20], we used a bi-directional MR to examine the effect of genetically predicted eGFR on serum testosterone.

## Methods

### Study design

We used univariable MR, as well as multivariable MR, to control for pleiotropic effects of SHBG where necessary [21]. We used a one-sample MR study to examine the role of genetically predicted testosterone in CKD and kidney function (indicated by albuminuria and eGFR), by applying testosterone-related, genome-wide significant SNPs in univariable MR and additionally including SHBG-related, genome-wide significant SNPs in multivariable MR, to the individual level outcome data in men and women separately in the UK Biobank, by far the largest cohort in Europeans [22]. For validation, we assessed the associations of genetically predicted testosterone with hemoglobin and HDL-cholesterol; a finding consistent with well-established effects in RCTs provides some validation of the genetic instrument. We also tested the role of genetically predicted eGFR in serum testosterone to check for the reverse association.

### The role of testosterone in CKD and eGFR

#### Data source

The genetic predictors for testosterone and their associations with CKD and kidney function were both obtained from the UK Biobank. The UK Biobank is a large, ongoing, prospective cohort study, with median follow up time of 11.1 years [22]. The UK Biobank recruited 502,713 people (aged 40–69 years, mean age 56.5 years, 45.6% men) from 2006 to 2010 from England, Scotland, and Wales; 94% were of self-reported European ancestry.

#### Genetic predictors for testosterone

MR is based on three assumptions, i.e., the genetic variants, specifically here single nucleotide polymorphisms (SNPs), are strongly related to the exposure (relevance), are not associated with confounders of the exposure-outcome relation (independence), and only influence the outcome via the exposure (exclusion-restriction) [23]. To meet these assumptions, we used genome-wide significant (*p* < 5 × 10^−8^) SNPs. The genetic instruments were obtained from the largest, sex-specific genome-wide association study (GWAS) conducted in the UK Biobank (178,782 white British men and 230,454 white British women) and replicated in three independent studies (CHARGE Consortium, Twins UK, and EPIC-Norfolk) [21]. The GWAS provided 125 independent SNPs (*r*^2^ < 0.05) for bioavailable testosterone in men and 254 independent SNPs for testosterone in women, with minor allele frequency > 0.1%, imputation quality score > 0.5, and little genetic correlation with SHBG (− 0.05 in men and − 0.06 in women) [21]. We checked for the Hardy-Weinburg equilibrium (HWE) and dropped any SNP with HWE *p* value < 10^−50^ [24]. The GWAS also provided 231 independent SNPs for total testosterone in men and 180 independent SNPs for bioavailable testosterone in women [21], which are highly correlated with SHBG [21]. As such, multivariable MR controlling for SHBG may have the greatest validity for these instruments, but for comprehensiveness, we also presented results of univariable MR for comparison.

To satisfy the assumption of independence, we checked for their association with potential confounders, including Townsend index, smoking, alcohol drinking, and physical activity in the UK Biobank summary statistics. We dropped SNPs associated with any of these potential confounders at genome-wide significance (*p* < 5 × 10^−8^). We also checked in PhenoScanner (www.phenoscanner.medschl.cam.ac.uk), a platform with comprehensive information on the associations of genotype and phenotype, to see whether these SNPs were associated with the potential confounders, and dropped SNP(s) associated with any of these potential confounders at genome-wide significance.

#### Genetic predictors for multivariable MR

Multivariable MR controlling for SHBG used genetic predictors for the exposure (bioavailable testosterone or total testosterone) and SHBG. The genetic predictors for testosterone were as used in univariable MR. The genetic predictors for SHBG were provided by the GWAS in the UK Biobank, with 357 SNPs in men and 359 SNPs in women [21]. After combining the genetic predictors for an exposure and SHBG, we dropped overlapping SNPs, correlated SNPs (*r*^2^ > 0.05), and SNPs whose correlations with other SNPs not available in MR-Base or LDlink (https://ldlink.nci.nih.gov/). The remaining SNPs were used for multivariable MR.

#### Genetic associations with CKD and kidney function

The genetic associations with CKD were obtained using individual-level, de-identified information from the UK Biobank (under application #42468). CKD events included mortality, morbidity, and self-reported chronic renal failure (ICD10-code: N18, ICD9-code: 5859, and self-report code: 1192); indications of renal replacement therapy (ICD-10 code Z99.2 (dependence on renal dialysis), Z94.0 (kidney transplant status), and Z49 (encounter for care involving renal dialysis)), identified based on a nurse-led interview at recruitment; record linkage to all health service encounters; deaths in the ongoing follow-up [22]; and eGFR, calculated based on the CKD-EPI formula using serum creatinine [25], less than 60 ml/min per 1.73 m^2^. Other outcomes included albuminuria and eGFR calculated based on the CKD-EPI formula using serum creatinine (eGFRcr) [25] and using both creatinine and cystatin C (eGFRcr_cys) [26].

In the UK Biobank, genotyping was assessed using two very similar arrays, i.e., the UK BiLEVE array and UK Biobank Axiom array. To control for population stratification, we restricted our analysis to participants with white British ancestry based on self-report and genetic quality control information. For quality control, we excluded participants with inconsistent self-reported and genotyped sex, or excess relatedness (more than 10 putative third-degree relatives) or abnormal sex chromosomes (such as XXY), or poor-quality genotyping (heterozygosity or missing rate > 1.5%). After quality control, we identified 179,916 white British men (6016 CKD cases) and 212,079 white British women (5958 CKD cases) updated till December 2019. Albuminuria was identified based on urine albumin-to-creatinine ratio (UACR) > 30 mg/g [27], where urine microalbumin was measured using an immune-turbidimetric assay, and urine creatinine was measured using an enzymatic assay (http://biobank.ndph.ox.ac.uk/showcase/showcase/docs/urine_assay.pdf); participants with microalbumin lower than the detection limit were considered to be free from albuminuria. We identified 8845 men and 8275 women with albuminuria. Logistic regression was used to obtain the association of each SNP with CKD and albuminuria, and linear regression was used for eGFR. We controlled for age, sex, 20 principal components, assay array, body mass index (BMI), systolic blood pressure (SBP), and diastolic blood pressure (DBP). BMI and blood pressure were controlled for because they are risk factors in common for various chronic diseases such as CKD and cardiovascular disease (CVD) [28, 29]; controlling for the common risk factors may partly address selection bias from competing risk from other diseases before recruitment [29, 30].

#### Genetic associations with hemoglobin and HDL-cholesterol

For validation, we examined the association of genetically predicted testosterone with hemoglobin and HDL-cholesterol as positive controls. The sex-specific associations of each SNP with hemoglobin and HDL-cholesterol were obtained from the UK Biobank summary statistics provided by Neale Lab (http://www.nealelab.is/uk-biobank/), using linear regression adjusted for age, age^2^, and 20 principal components in the UK Biobank.

### The role of kidney function in serum testosterone

#### Genetic predictors for eGFR

To meet the MR assumptions, we used genetic predictors reaching genome-wide significance (*p* < 5 × 10^−8^) in the recent GWAS meta-analysis in CKDGen [31]. We selected independent SNPs (*r*^2^ < 0.01) predicting eGFR using the “clumping” function of MR-Base. Given it is a two-sample MR, we also checked for palindromic SNPs (i.e., SNPs with alleles (A/T and C/G) that pair with each other) and aligned on effect allele frequency. We dropped palindromic SNPs if the minor allele frequency was close to 0.50, such as 0.48 to 0.52, which are difficult to align unequivocally.

#### Genetic association with serum testosterone

We obtained the association of each eGFR-related SNP with serum testosterone from the UK Biobank summary statistics, provided by Neale Lab (http://www.nealelab.is/uk-biobank/), in 167,020 white British men and 194,174 women, adjusted for age, age^2^, and 20 principal components. If the SNPs predicting eGFR were not in the UK Biobank, proxy SNPs (*r*^2^ ≥ 0.8), identified from the LDlink (https://ldlink.nci.nih.gov/), were used.

### Statistical analysis for MR estimates using univariable MR

MR estimates were derived from the Wald estimates (genetic association with outcome divided by the genetic association with the exposure), i.e., genetic association with CKD, albuminuria, or eGFR divided by the genetic association with testosterone when assessing the renal effect of testosterone, and genetic association with testosterone divided by the genetic association with eGFR when assessing the role of kidney function in testosterone. We obtained the Wald estimate for each SNP and then meta-analyzed them using inverse variance weighting (IVW) with multiplicative random effects. To examine whether the MR estimates were larger in men than in women, we calculated the *p* value for the differences in sex-specific estimates (log odds ratios for CKD and albuminuria, and beta-coefficients for eGFR), where we used a well-established formula to calculate the *z*-statistic, and then obtained the one-tailed *p* value [32].

In sensitivity analysis, we used other statistical methods, specifically a weighted median [33] and Mendelian randomization pleiotropy residual sum and outlier (MR-PRESSO) [34]. The weighted median estimate is robust to invalid instruments and able to provide consistent estimation even when up to 50% of the weight is from invalid SNPs [33]. MR-PRESSO is able to identify outliers with potential horizontal pleiotropy amongst multiple genetic variants and provide a corrected estimate after removing these outliers [34]. If MR-PRESSO identified outlier(s), we used the corrected estimates from MR-PRESSO instead of IVW in the main analysis.

Power calculations were conducted where the sample size needed for MR is approximately the sample size for the conventional observational study divided by the variance in the exposure explained by the SNPs [35]. Specifically, the variance explained by each SNP was calculated using beta^2^ × 2 × minor allele frequency (MAF) × (1 − MAF), where beta is the standardized beta coefficient for the effect allele and MAF is the minor allele frequency [36]. For CKD events, the required sample size was calculated based on the log odds ratio, the ratio of cases to non-cases, and the variance explained by the SNPs [37]. For eGFR, the continuous outcome, it was calculated based on the effect size and the variance explained by the SNPs [35].

### Statistical analysis for MR estimates using multivariable MR

Multivariable MR is an extension of conventional univariable MR. It takes advantage of pleiotropic SNPs predicting two or more exposures and can be used to estimate the effect of one exposure after controlling for the other exposure(s) [38], here the effect of testosterone on CKD and kidney function after controlling for SHBG. In multivariable MR, we used MR Egger to detect whether the genetic predictors were acting other than via testosterone or SHBG (directional pleiotropy) indicated by a non-zero intercept [39], in which case we used multivariable MR Egger estimates in the main analysis.

All statistical analyses were conducted using R version 3.6.2 (R Foundation for Statistical Computing, Vienna, Austria), the “clumping” function of MR-Base, and the R package “MendelianRandomization.”

### Patient and public involvement statement

Patients or the public were not involved in the design, or conduct, or reporting, or dissemination plans of our research.

### Ethical approval

This research has been conducted using the UK Biobank Resource under application number 42468. No original data were collected for the MR study. Ethical approval for each of the studies included in the investigation can be found in the original publications (including informed consent from each participant). The UK Biobank has already received ethical approval from the Research Ethics Committee, and participants provided written informed consent. The analysis of other publicly available summary statistics does not require additional ethical approval.

## Results

### The role of testosterone in CKD and kidney function

We used the previously published 125 genome-wide significant SNPs for bioavailable testosterone in men and 254 SNPs for total testosterone in women. No SNP violated HWE. One SNP in men and 2 SNPs in women were related to alcohol drinking. Given the unclear causal role of alcohol in CKD, we kept these SNPs in the main analysis but excluded them in the sensitivity analysis. Of these SNPs, 6 SNPs in men and 2 SNPs in women were not available in the summary statistics from Neale Lab (http://www.nealelab.is/uk-biobank/), nor were their proxies, so 119 SNPs in men and 252 SNPs in women were used for hemoglobin and HDL-cholesterol, whereas all SNPs were used for CKD and kidney function. Similarly, we used the previously published 231 genome-wide significant SNPs for total testosterone in men and the 180 SNPs for bioavailable testosterone in women. All SNPs were used for CKD and kidney function; 224 SNPs in men and 176 SNPs in women were used for hemoglobin and HDL-cholesterol.

In multivariable MR for bioavailable testosterone controlling for SHBG in men, there were 125 SNPs for bioavailable testosterone and 357 SNPs for SHBG (in total 482 SNPs). After excluding 3 duplicate SNPs, 8 correlated SNPs, and 7 SNPs with unclear correlation information, 464 SNPs remained and were used. In women, there were 180 SNPs for bioavailable testosterone and 359 SNPs for SHBG (in total 539 SNPs). After excluding 26 duplicate SNPs, 47 correlated SNPs, and 3 SNPs with unclear correlation information, 463 SNPs remained. In multivariable MR for total testosterone controlling for SHBG in men, there were 231 SNPs for total testosterone and 357 SNPs for SHBG (in total 588 SNPs). After excluding 39 duplicate SNPs, 87 correlated SNPs, and 2 SNPs with unclear correlation information, 460 SNPs remained and were used. In women, there were 254 SNPs for total testosterone and 359 SNPs for SHBG (in total 613 SNPs). After excluding 2 duplicate SNPs, 29 correlated SNPs, and 1 SNPs with unclear correlation information, 581 SNPs remained.

Using univariable MR, genetically predicted bioavailable testosterone was associated with higher risk of CKD and albuminuria, and lower eGFRcr and eGFRcr_cys in men, but had null associations in women (Table 1), whilst neither bioavailable nor total testosterone was related to CKD or kidney function in women (Table 1 and Additional file 1: Table S1). The difference by sex was significant for CKD and albuminuria (*p* values for sex difference 0.04 and 0.01, respectively) as well as for eGFR (*p* values for sex difference less than 1 × 10^−5^ for both eGFRcr and eGFRcr_cys). The associations were robust to different methods (Additional file 1: Table S2) and to excluding SNPs related to alcohol drinking (Additional file 1: Table S3). Similar patterns of associations were found in multivariable MR (Table 2). Multivariable MR Egger did not indicate directional pleiotropy except for bioavailable testosterone and CKD in women (Additional file 1: Table S4). At an approximate *R*^2^ of 0.056 in men and of 0.085 in women, the UK Biobank has 0.8 power to detect an odds ratio (OR) of about 1.17 for CKD and 1.14 for albuminuria per one standard deviation increase in testosterone in men and an OR of about 1.14 for CKD and 1.12 for albuminuria in women [37], as well as an effect size of about 0.03 standard deviations for eGFR in men and of 0.02 in women [35]. Genetically predicted bioavailable testosterone in men and women, as well as genetically predicted total testosterone in women, showed the associations expected from meta-analysis of RCTs [19], i.e., a positive association with hemoglobin and inverse association with HDL-cholesterol (Table 3), whist total testosterone in men was related to higher HDL-cholesterol in univariable MR but was as expected, related to lower HDL-cholesterol, in multivariable MR controlling for SHBG (Additional file 1: Table S5).

**Table 1:** Associations of genetically predicted bioavailable testosterone in men and of genetically predicted total testosterone in women with CKD and kidney function using univariable Mendelian randomization in the UK Biobank

Outcome	Exposure	#SNPs	Sex	OR	95% CI	*p*
CKD	Genetically predicted bioT	125	Men	**1.17**	**1.03, 1.33**	**0.01**
	Genetically predicted TT	254	Women	1.02	0.92, 1.14	0.68
Albuminuria	Genetically predicted bioT	125	Men	1.15^*^	**1.04, 1.27**	**0.008**
	Genetically predicted TT	254	Women	0.96	0.88, 1.06	0.43
				Beta	95% CI	*p*
eGFR_cr (ml/min per 1.73 m^2^)	Genetically predicted bioT	125	Men	− 1.70^*^	**− 2.09, − 1.31**	**2.4 × 10**^**−14**^
	Genetically predicted TT	254	Women	− 0.24^*^	− 0.49, 0.02	0.07
eGFR_crcys (ml/min per 1.73 m^2^)	Genetically predicted bioT	125	Men	− 1.27^*^	**− 1.62, − 0.91**	**1.0 × 10**^**−10**^
	Genetically predicted TT	254	Women	0.12^*^	− 0.14, 0.39	0.36

*bioT* bioavailable testosterone, *CKD* chronic kidney disease, *eGFR* estimated glomerular filtration rate, *TT* total testosterone

^*^Mendelian randomization pleiotropy residual sum and outlier (MR-PRESSO) was used for the analysis; otherwise, inverse variance weighting was used

**Table 2:** Associations of bioavailable testosterone and total testosterone with CKD and kidney function in the UK Biobank using multivariable MR

Exposure	Outcome	Sex	#SNPs	OR	95% CI	*p*
Genetically predicted bioavailable testosterone	CKD	Men	464	**1.14**	**1.02, 1.28**	**0.025**
		Women	463	0.92	0.71, 1.19	0.52
	Albuminuria	Men	464	**1.17**	**1.04, 1.32**	**0.01**
		Women	463	0.85	0.70, 1.04	0.12
				Beta	95% CI	*p*
	eGFR_cr (ml/min per 1.73 m^2^)	Men	464	**− 1.06**	**− 1.52, − 0.60**	**6.7 × 10**^**−6**^
		Women	463	− 0.71	− 1.70, 0.28	0.16
	eGFR_crcys (ml/min per 1.73 m^2^)	Men	464	− 0.48	− 0.97, 0.005	0.05
		Women	463	− 0.58	− 1.63, 0.47	0.28
Genetically predicted total testosterone	CKD	Men	460	1.13	0.96, 1.32	0.13
		Women	581	1.00	0.90, 1.11	0.96
	Albuminuria	Men	460	**1.19**	**1.02, 1.39**	**0.03**
		Women	581	0.95	0.87, 1.04	0.30
				Beta	95% CI	*p*
	eGFR_cr (ml/min per 1.73 m^2^)	Men	460	**− 1.20**	**− 1.80, − 0.61**	**7.7 × 10**^**−5**^
		Women	581	− 0.31	− 0.75, 0.12	0.16
	eGFR_crcys (ml/min per 1.73 m^2^)	Men	460	**− 0.66**	**− 1.30, − 0.02**	**0.045**
		Women	581	− 0.12	− 0.60, 0.36	0.62

*CKD* chronic kidney disease, *eGFR* estimated glomerular filtration rate

Multivariable MR controlling for sex hormone binding globulin was used

**Table 3:** Associations of genetically predicted bioavailable testosterone in men and of genetically predicted total testosterone in women with hemoglobin and HDL-cholesterol for validation of the genetic instrument in the UK Biobank

Outcome	Exposure	#SNPs	Sex	Methods	Beta	95% CI	*p*
Hemoglobin	Genetically predicted bioT	119	Men	MR-PRESSO	**0.30**	**0.26, 0.33**	**8.3 × 10**^**−31**^
				WM	**0.33**	**0.28, 0.37**	**2.0 × 10**^**−48**^
	Genetically predicted TT	252	Women	MR-PRESSO	**0.05**	**0.03, 0.08**	**8.6 × 10**^**−5**^
				WM	0.03	− 0.002, 0.06	0.07
HDL-cholesterol	Genetically predicted bioT	119	Men	MR-PRESSO	**− 0.07**	**− 0.10, − 0.04**	**4.0 × 10**^**−5**^
				WM	**− 0.08**	**− 0.12, − 0.04**	**4.1 × 10**^**−5**^
	Genetically predicted TT	252	Women	MR-PRESSO	**− 0.06**	**− 0.09, − 0.04**	**1.2 × 10**^**−6**^
				WM	**− 0.05**	**− 0.09, − 0.02**	**0.003**

*HDL* high-density lipoprotein, *IVW* inverse variance weighting, *WM* weighted median, *MR-PRESSO* Mendelian randomization pleiotropy residual sum and outlier

### The role of kidney function in testosterone

There were 264 SNPs for eGFR in the GWAS meta-analysis [31]. Of the 264 SNPs, 228 were uncorrelated. After excluding 1 SNP not available for genetic association with testosterone and 4 SNPs that are palindromic with minor allele frequency close to 0.5, 223 SNPs were used (Additional file 1: Table S6). Genetically predicted eGFR was not related to testosterone in men or women (Table 4). The associations were robust to different analysis methods (Table 4).

**Table 4:** Association of genetically predicted eGFR with serum testosterone using Mendelian randomization in the UK Biobank

Exposure	Outcome	#SNPs	Sex	Methods	Beta	95% CI	*p*
Genetically predicted eGFR	Serum testosterone	223	Men	MR-PRESSO	0.12^*^	− 0.12, 0.37	0.33
				WM	0.02	− 0.27, 0.32	0.89
			Women	MR-PRESSO	0.06^*^	− 0.18, 0.30	0.63
				WM	0.09	− 0.23, 0.41	0.57

## Discussion

In an MR study using univariable and multivariable MR, our study for the first time suggests testosterone affects CKD and kidney function in a sex-specific way. Testosterone might be an underlying cause of CKD in men but not in women. Meanwhile, genetically predicted kidney function did not affect serum testosterone. Our finding may be relevant to the sex disparity in CKD and kidney function, consistent with the sex-specific role of testosterone in CVD [40], and with expectations from evolutionary biology [4–6].

Our findings did not corroborate a beneficial association of testosterone with kidney function sometimes seen in observational studies [15, 16], possibly due to poor health lowering both testosterone and kidney function [41, 42]. However, dietary factors, such as a high protein diet, which boost testosterone in men [43], increase CKD. In contrast, some medications lowering testosterone show a benefit for kidney function. For example, statins lower testosterone [44] and can slow down the progression of CKD [45]. Although the benefits may be due to lipid lowering, recent MR studies do not support a role of LDL-cholesterol in CKD [46]. Our findings are also consistent with changing disease patterns with economic development. Improvements in living conditions that enable higher levels of testosterone [47], with corresponding effects on kidney function, may be relevant to the rising rates of CKD that emerge with economic development, such as in China [1].

To our knowledge, this MR study is the first to examine the role of testosterone in CKD and kidney function. Despite the novelty, our study has several limitations. First, MR relies on three assumptions, i.e., relevance, independence, and exclusion-restriction [23]. To satisfy these assumptions, we used genetic variants from the largest sex-specific published GWAS of testosterone based on the UK Biobank and with replication in three independent studies [21]. We also checked the randomization from their associations with potential confounders, such as socioeconomic position and lifestyle. To address the assumption of exclusion-restriction, we tested and corrected for potential pleiotropy using MR-PRESSO. We also used multivariable MR to control for pleiotropic associations with SHBG. Second, measurement error might exist in the classification of CKD or the single time-point assay of testosterone. However, any measurement error should be non-differential, thus bias toward a null association, which should not affect the directions of associations. Third, our study could be affected by survivor bias [30] and by competing risk, i.e., by an event whose occurrence precludes the occurrence of CKD [29]. For example, if testosterone leads to death from ischemic heart disease at 74 years [48], death from CKD which usually occurs at 85 years will not be observed [49]. As such, the potential harm for CKD will be underestimated due to competing events. To control for the bias arising from competing risk, we adjusted for common causes of CKD and CVD [29], specifically BMI, smoking, and blood pressure. Fourth, the participants in the UK Biobank are healthier than the general population [50], and the majority do not suffer from CKD, so the estimates may not be applicable to CKD patients; however, the directions of associations should be consistent. Fifth, the associations in Europeans may not apply to other populations, such as Asians. However, causal effects should be consistent across settings, for example the effect on HDL-cholesterol was also reported in our previous MR study in Chinese [51]. Nevertheless, the effect size might vary by population according to testosterone levels; as such, replication in Asians would be worthwhile. Sixth, the genetic effects might be buffered by compensatory processes or feedback mechanisms. Such compensation would be expected to mitigate the genetic effects, thus biasing toward the null. As such, the estimates for CKD and kidney function might be underestimated. Finally, the wide age range in the UK Biobank participants may increase the variation in testosterone levels. However, this will only lower the precision of the testosterone GWAS and MR estimates, rather than affect the directions of association.

Despite the consistency with evolutionary biology [4–6], the known sex disparity [3], and evidence from animal experiments [9, 10], these novel findings need to be interpreted cautiously. First, the association with CKD needs to be replicated in a larger sample with more power. Second, due to the limited number with follow-up measures of kidney function, we used baseline rather than decline in kidney function. It would be valuable to assess the role of testosterone in CKD progression, specifically the relevance to decline in kidney function, when sufficient follow-up data in the UK Biobank and suitable analytic techniques are available [52]. Third, the role of endogenous testosterone might be different from the role of testosterone supplementation or other lifestyle factors that modulate testosterone. However, the findings for hemoglobin and HDL-cholesterol in MR showed consistency with meta-analysis of RCTs [19]. Moreover, MR estimates lifetime effects rather than the effects of a short-term exposure. As such, the effect on CKD and kidney function might not be comparable to the acute renal effect of testosterone supplementation, although the use of testosterone gel lowers eGFR in RCT [14]. Finally, the underlying pathways from testosterone to kidney function remain to be clarified. The inverse association with eGFRcr might be due to or partly due to testosterone increasing muscle mass and thereby increasing serum creatinine. However, the inverse association remained for eGFRcr_cys. The similar pattern of associations for CKD and albuminuria also provides consistent evidence concerning the renal effects of testosterone. The effect size is small and might not be clinically significant; however, an MR study is more useful in determining the direction of causation than the magnitude of an effect size [53]. Moreover, a small effect size may be highly relevant at the population level [54]. Several mechanisms might underlie the renal effects of testosterone, including an effect on cellular apoptosis that may impact renal disease progression, an effect on glomerular matrix accumulation, an influence on the synthesis and activity of several cytokines and vasoactive agents, an interaction with the renin-angiotensin system [55], an increase in the generation of reactive oxygen species [55, 56], and a pro-inflammatory effect in the kidney [57]. Inflammation and immune function may underlie the pathology of CKD [58], and testosterone is known to be immunomodulatory [59]. In a clinical case report, testosterone directly modulated kidney perfusion [13], but the specific pathway is unclear. Clarifying these pathways, especially as regards any sex differences in the response to testosterone, such as immune function [59], would be valuable.

From the perspective of clinical and public health practice, our findings suggest that testosterone and drivers of testosterone are potential targets for lowering the burden of kidney disease in men rather than in women, consistent with the dominant role of testosterone in men’s reproduction and health [4]. Medications or lifestyle factors that modulate testosterone might be effective for the prevention and treatment of CKD and may be expected to play a sex-specific effect. As such, our study highlights the importance of considering sex-specific causes and treatments for CKD and raises the question as to whether any causes of CKD specific to women exist. Exploration of such factors and the underlying pathways would give insights to the re-positioning of existing drugs, new drug development, and lifestyle recommendations.

## Conclusions

Genetically predicted testosterone was associated with CKD and unfavorable kidney function in men, which might contribute to the sex disparity in CKD. Serum testosterone was not affected by kidney function. Clarifying the underlying pathways could provide new insights for prevention and treatment strategies in both men and women. Replication in other studies, especially in understudied populations, is needed.

## Supplementary information

**Additional file 1: Table S1.** Associations of genetically predicted total testosterone in men and of genetically predicted bioavailable testosterone in women with CKD and kidney function in the UK Biobank using univariable MR. **Table S2.** Sensitivity analysis on the associations of genetically predicted bioavailable testosterone in men and of genetically predicted total testosterone in women with CKD and kidney function using different analysis methods in the univariable Mendelian randomization in the UK Biobank. **Table S3.** Sensitivity analysis excluding genetic variants^*^ related to alcohol in Mendelian randomization in the UK Biobank. **Table S4.** Sensitivity analysis on the associations of bioavailable testosterone and total testosterone with CKD and kidney function in men and women in the UK Biobank using multivariable MR Egger. **Table S5.** Associations of genetically predicted total testosterone in men and of genetically predicted bioavailable testosterone in women with hemoglobin and HDL-cholesterol for validation of the genetic instrument in the UK Biobank using univariable and multivariable MR^*^. **Table S6.** Genetic predictors for eGFR.

## Data Availability

The access of data from the UK Biobank can be obtained by application to the UK Biobank (http://biobank.ctsu.ox.ac.uk/crystal/). The summary statistics can be downloaded from the website (http://www.nealelab.is/uk-biobank/).

## Abbreviations

BMI: Body mass index
CVD: Cardiovascular disease
CKD: Chronic kidney disease
DBP: Diastolic blood pressure
eGFR: Estimated glomerular filtration rate
GWAS: Genome-wide association study
HDL: High-density lipoprotein
HWE: Hardy-Weinburg equilibrium
IVW: Inverse variance weighting
MAF: Minor allele frequency
MR: Mendelian randomization
MR-PRESSO: Mendelian randomization pleiotropy residual sum and outlier
OR: Odds ratio
RCT: Randomized controlled trial
SBP: Systolic blood pressure
SHBG: Sex hormone binding globulin
SNP: Single nucleotide polymorphism
UACR: Urine albumin-to-creatinine ratio
