# Supplementary material for: The role of testosterone in chronic kidney disease and kidney function in men and women: a bi-directional Mendelian randomization study in the UK Biobank
Source: BMC Med. 2020 Jun 4;18:122. doi: 10.1186/s12916-020-01594-x (PMC7271464; doi:10.1186/s12916-020-01594-x)
Supplement: Supplementary file 1 — Additional file 1: Table S1. Associations of genetically predicted total testosterone in men and of genetically predicted bioavailable testosterone in women with CKD and kidney function in the UK Biobank using univariable MR. Table S2. Sensitivity analysis on the associations of genetically predicted bioavailable testosterone in men and of genetically predicted total testosterone in women with CKD and kidney function using different analysis methods in the univariable Mendelian randomization in the UK Biobank. Table S3. Sensitivity analysis excluding genetic variants* related to alcohol in Mendelian randomization in the UK Biobank. Table S4. Sensitivity analysis on the associations of bioavailable testosterone and total testosterone with CKD and kidney function in men and women in the UK Biobank using multivariable MR Egger. Table S5. Associations of genetically predicted total testosterone in men and of genetically predicted bioavailable testosterone in women with hemoglobin and HDL-cholesterol for validation of the genetic instrument in the UK Biobank using univariable and multivariable MR*. Table S6. Genetic predictors for eGFR. [file 12916_2020_1594_MOESM1_ESM.pdf]

Table S1. Associations of genetically predicted total testosterone in men and of genetically predicted bioavailable testosterone in women with CKD and kidney function in the UK Biobank using univariable MR

Outcome	Exposure	#SNPs	Sex	OR	95% CI	*p*
CKD	Genetically predicted TT	231	Men	0.94	0.85, 1.04	0.22
	Genetically predicted bioT	180	Women	0.96	0.82, 1.11	0.55
Albuminuria	Genetically predicted TT	231	Men	1.02	0.93, 1.12	0.71
	Genetically predicted bioT	180	Women	1.01	0.88, 1.15	0.94
				Beta	95% CI	*p*
eGFR_cr (ml/min per 1.73 m^2)	Genetically predicted TT	231	Men	-0.26	-0.61, 0.09	0.15
	Genetically predicted bioT	180	Women	-0.09	-0.64, 0.47	0.76
eGFR_crcys (ml/min per 1.73 m^2)	Genetically predicted TT	231	Men	-0.23	-0.62, 0.16	0.25
	Genetically predicted bioT	180	Women	0.06	-0.61, 0.74	0.86

bioT, bioavailable testosterone; CKD, chronic kidney disease; eGFR, estimated glomerular filtration rate; TT, total testosterone. Inverse variance weighting was used.

Table S2. Sensitivity analysis on the associations of genetically predicted bioavailable testosterone in men and of genetically predicted total testosterone in women with CKD and kidney function using different analysis methods in the univariable Mendelian randomization in the UK Biobank

Outcome	Exposure	#SNPs	Sex	Methods	OR	95% CI	*p*
CKD	Genetically predicted bioT	125	Men	WM	**1.36**	**1.12, 1.65**	**0.002**
				MR PRESSO	**1.17**	**1.03, 1.33**	**0.01**
	Genetically predicted TT	254	Women	WM	1.08	0.89, 1.32	0.44
Albuminuria	Genetically predicted bioT	125	Men	WM	**1.28**	**1.09, 1.51**	**0.002**
	Genetically predicted TT	254	Women	WM	0.92	0.77, 1.09	0.33
				MR PRESSO	0.96	0.88, 1.06	0.43
					Beta	95% CI	*p*
eGFR_cr (ml/min per 1.73 m^2)	Genetically predicted bioT	125	Men	WM	**-2.08**	**-2.45, -1.70**	**3.5×10^{-27}**
	Genetically predicted TT	254	Women	WM	-0.15	-0.53, 0.22	0.43
eGFR_crcys (ml/min per 1.73 m^2)	Genetically predicted bioT	125	Men	WM	**-1.38**	**-1.78, -0.98**	**1.8×10^{-11}**
	Genetically predicted TT	254	Women	WM	0.03	-0.38, 0.45	0.87

bioT, bioavailable testosterone; CKD, chronic kidney disease; eGFR, estimated glomerular filtration rate; TT, total testosterone; WM, weighted median; MR-PRESSO, Mendelian Randomization Pleiotropy Residual Sum and Outlier

Table S3. Sensitivity analysis excluding genetic variants* related to alcohol in Mendelian randomization in the UK Biobank

Outcome	Exposure	#SNPs	Sex	OR	95% CI	*p*
CKD	Genetically predicted bioT	124	Men	**1.17**	**1.04, 1.33**	**0.01**
	Genetically predicted TT	252	Women	1.01	0.91, 1.13	0.84
Albuminuria	Genetically predicted bioT	124	Men	**1.15**	**1.04, 1.27**	**0.008**
	Genetically predicted TT	252	Women	0.97	0.89, 1.07	0.58
				Beta	95% CI	*p*
eGFR_cr (ml/min per 1.73 m^2)	Genetically predicted bioT	124	Men	**-1.64**	**-2.03, -1.25**	**1.4×10^{-13}**
	Genetically predicted TT	252	Women	-0.22	-0.48, 0.04	0.09
eGFR_crcys (ml/min per 1.73 m^2)	Genetically predicted bioT	124	Men	**-1.25**	**-1.61, -0.90**	**1.9×10^{-10}**
	Genetically predicted TT	252	Women	0.13	-0.14, 0.40	0.34

bioT, bioavailable testosterone; CKD, chronic kidney disease; eGFR, estimated glomerular filtration rate; TT, total testosterone. MR-PRESSO (Mendelian Randomization Pleiotropy Residual Sum and Outlier) was used for the analysis on albuminuria in men, and eGFR_cr and eGFR_crcys in men and women, otherwise inverse variance weighting was used.

*One SNP (rs8061590) in men and two SNPs (rs1260326 and rs1229984) in women were excluded.

Table S4. Sensitivity analysis on the associations of bioavailable testosterone and total testosterone with CKD and kidney function in men and women in the UK Biobank using multivariable MR Egger

Exposure	Outcome	Sex	#SNPs	OR	95% CI	*p*	*Intercept p*
Genetically predicted bioavailable testosterone	CKD	Men	464	1.12	0.98, 1.28	0.10	0.62
		Women	463	0.92	0.71, 1.19	0.52	0.03
	Albuminuria	Men	464	**1.17**	**1.02, 1.34**	**0.03**	0.92
		Women	463	0.82	0.65, 1.03	0.09	0.49
				Beta	95% CI	*p*	
	eGFR_cr (ml/min per 1.73 m^2)	Men	464	**-1.10**	**-1.64, -0.56**	**7.0×10^{-5}**	0.82
		Women	463	-0.23	-1.36, 0.90	0.69	0.11
	eGFR_crcys (ml/min per 1.73 m^2)	Men	464	**-0.59**	**-1.17, -0.02**	**0.04**	0.51
		Women	463	-0.16	-1.36, 1.04	0.79	0.22
			#SNPs	OR	95% CI	*p*	
Genetically predicted total testosterone	CKD	Men	460	1.16	0.97, 1.39	0.10	0.50
		Women	581	1.06	0.92, 1.21	0.44	0.23
	Albuminuria	Men	460	1.19	0.99, 1.42	0.06	0.89
		Women	581	0.98	0.87, 1.11	0.81	0.43
				Beta	95% CI	*p*	
	eGFR_cr (ml/min per 1.73 m^2)	Men	460	**-1.28**	**-1.96, -0.59**	**0.0002**	0.66
		Women	581	-0.47	-1.03, 0.10	0.11	0.41
	eGFR_crcys (ml/min per 1.73 m^2)	Men	460	-0.49	-1.23, 0.24	0.19	0.36
		Women	581	-0.10	-0.72, 0.53	0.77	0.94

CKD, chronic kidney disease; eGFR, estimated glomerular filtration rate

*Multivariable MR controlling for sex hormone-binding globulin was used.

Table S5. Associations of genetically predicted total testosterone in men and of genetically predicted bioavailable testosterone in women with hemoglobin and HDL-cholesterol for validation of the genetic instrument in the UK Biobank using univariable and multivariable MR*

Outcome	Exposure	Sex	Methods	Beta	95% CI	*p*	MVMR Egger intercept *p*
Hemoglobin	Genetically predicted TT	Men	Univariable MR IVW	**0.16**	**0.12, 0.21**	**2.2×10^{-12}**	
			MVMR IVW	**0.35**	**0.28, 0.41**	**4.0×10^{-28}**	
			MVMR Egger	**0.32**	**0.25, 0.39**	**8.1×10^{-19}**	0.11
	Genetically predicted bioT	Women	Univariable MR IVW	**0.12**	**0.06, 0.17**	**6.4×10^{-5}**	
			MVMR IVW	**0.12**	**0.04, 0.21**	**0.004**	
			MVMR Egger	0.07	-0.02, 0.16	0.15	0.01
HDL-cholesterol	Genetically predicted TT	Men	Univariable MR IVW	**0.08**	**0.04, 0.12**	**0.0003**	
			MVMR IVW	0.01	-0.07, 0.08	0.88	
			MVMR Egger	**-0.12**	**-0.19, -0.04**	**0.003**	<0.001
	Genetically predicted bioT	Women	Univariable MR IVW	**-0.21**	**-0.28, -0.15**	**2.8×10^{-10}**	
			MVMR IVW	-0.10	-0.21, 0.01	0.08	
			MVMR Egger	0.08	-0.04, 0.20	0.20	<0.001

bioT, bioavailable testosterone; TT, total testosterone HDL, high density lipoprotein. IVW, inverse variance weighting; MVMR, multivariable Mendelian randomization

*Multivariable MR controlling for sex hormone-binding globulin was used.

Table S6. Genetic predictors for eGFR

SNP	Effect allele	Beta	SE	P value
rs11166440	A	0.002	3.00E-04	1.80E-10
rs10857788	A	0.003	4.00E-04	2.00E-16
rs12736457	C	0.005	5.00E-04	1.00E-25
rs267738	T	-0.005	4.00E-04	1.20E-32
rs4971100	A	0.002	3.00E-04	8.30E-10
rs10159261	T	-0.003	3.00E-04	4.80E-25
rs3845534	A	-0.002	3.00E-04	1.20E-09
rs4656220	T	0.002	3.00E-04	3.30E-10
rs1011731	A	-0.002	3.00E-04	5.30E-09
rs3795503	T	0.002	3.00E-04	9.80E-10
rs78444298	A	-0.011	0.0014	4.90E-14
rs78329830	A	-0.005	9.00E-04	6.70E-09
rs12061708	A	-0.003	3.00E-04	9.60E-14
rs3850625	A	0.005	5.00E-04	1.10E-18
rs75625374	C	0.005	7.00E-04	4.50E-10
rs7535253	T	0.002	4.00E-04	1.30E-09
rs2577134	T	0.002	3.00E-04	1.50E-08
rs61830291	A	-0.004	6.00E-04	1.20E-09
rs417237	T	0.002	3.00E-04	7.50E-09
rs2749153	A	-0.003	3.00E-04	7.80E-23
rs2490391	A	-0.002	3.00E-04	1.30E-14
rs659437	T	-0.003	4.00E-04	3.30E-12
rs688540	A	-0.003	5.00E-04	3.00E-08
rs17413465	A	0.003	4.00E-04	8.90E-09
rs1757915	A	0.002	3.00E-04	2.90E-10
rs679843	T	0.002	3.00E-04	5.20E-10
rs1887252	C	-0.002	3.00E-04	2.90E-09
rs7543734	C	0.003	5.00E-04	9.90E-11
rs284859	T	0.003	4.00E-04	5.00E-12
rs1055256	A	0.003	3.00E-04	3.60E-16
rs6481598	C	0.002	4.00E-04	1.50E-09
rs7072591	A	0.002	3.00E-04	2.70E-09
rs8474	C	0.002	3.00E-04	1.80E-09
rs10821905	A	0.004	4.00E-04	9.40E-19
rs10821944	T	0.002	3.00E-04	3.90E-09
rs7475348	T	0.003	3.00E-04	1.20E-22
rs12240572	A	-0.003	6.00E-04	5.30E-09
rs816850	C	-0.002	4.00E-04	7.40E-09
rs9420446	T	0.002	4.00E-04	5.00E-08
rs80282103	A	0.008	6.00E-04	1.20E-44
rs2068888	A	-0.002	3.00E-04	4.40E-15
rs4918943	A	-0.002	4.00E-04	9.20E-09
rs6589750	A	0.002	3.00E-04	1.50E-09
rs10790452	T	0.002	3.00E-04	7.10E-09
rs11564722	T	0.003	4.00E-04	2.10E-20
rs63934	A	0.004	4.00E-04	3.90E-23
rs963837	T	-0.006	3.00E-04	4.30E-73
rs61897431	T	0.003	4.00E-04	6.30E-16
rs1541937	A	-0.003	4.00E-04	5.20E-17
rs1783827	A	-0.002	3.00E-04	3.80E-09
rs948493	T	-0.003	3.00E-04	2.00E-24
rs3892895	A	-0.002	3.00E-04	6.20E-13
rs11237450	A	0.003	4.00E-04	1.50E-14
rs117113238	A	0.004	6.00E-04	8.60E-11
rs10846157	A	-0.003	4.00E-04	8.30E-21
rs632887	A	0.003	3.00E-04	2.20E-24
rs11062167	A	-0.004	3.00E-04	2.50E-34
rs4238020	T	0.003	5.00E-04	8.40E-09
rs2634675	A	0.003	3.00E-04	2.70E-13
rs12313306	T	0.003	4.00E-04	1.50E-14

rs1275609	A	0.002	3.00E-04	4.60E-13
rs41284816	T	-0.008	0.0012	1.70E-10
rs500830	T	0.003	3.00E-04	2.00E-19
rs61993680	A	-0.002	3.00E-04	1.50E-08
rs72683923	T	-0.007	0.0013	3.40E-08
rs6574652	T	-0.002	3.00E-04	2.40E-08
rs1028455	A	0.002	3.00E-04	4.80E-10
rs17184313	T	-0.003	5.00E-04	2.00E-10
rs12913015	T	0.003	3.00E-04	2.30E-17
rs6492982	T	-0.003	4.00E-04	3.10E-20
rs1145077	T	-0.009	3.00E-04	6.90E-142
rs690428	A	-0.004	3.00E-04	8.20E-32
rs1994887	A	-0.002	4.00E-04	1.60E-08
rs956006	T	0.002	3.00E-04	4.40E-09
rs11071738	T	-0.003	3.00E-04	2.00E-15
rs351237	A	-0.002	3.00E-04	4.40E-08
rs4886696	A	-0.003	4.00E-04	2.00E-19
rs4886755	A	0.004	3.00E-04	2.00E-39
rs17507300	A	0.002	4.00E-04	1.00E-08
rs59646751	T	-0.002	3.00E-04	3.10E-12
rs193538	T	-0.002	3.00E-04	1.80E-09
rs438339	T	0.004	6.00E-04	5.00E-08
rs77924615	A	0.010	4.00E-04	1.50E-138
rs1635404	T	-0.003	4.00E-04	5.70E-11
rs9932625	A	-0.003	3.00E-04	2.20E-17
rs7203398	A	0.003	3.00E-04	4.70E-13
rs7185391	T	-0.003	4.00E-04	8.60E-14
rs62050038	A	0.003	4.00E-04	1.30E-11
rs1858800	T	0.002	3.00E-04	2.10E-09
rs28581385	A	-0.003	4.00E-04	1.40E-11
rs154656	A	-0.003	3.00E-04	4.10E-18
rs28735420	T	0.004	6.00E-04	8.80E-10
rs2349648	T	-0.002	3.00E-04	4.90E-08
rs9891340	T	0.002	4.00E-04	2.70E-11
rs2440165	T	0.004	3.00E-04	1.70E-31
rs2411192	A	-0.002	3.00E-04	2.10E-15
rs4794813	A	0.006	4.00E-04	3.60E-53
rs227731	T	0.002	3.00E-04	1.40E-08
rs9903801	C	0.005	4.00E-04	1.00E-27
rs9895661	T	0.007	4.00E-04	2.50E-71
rs8866	C	-0.002	3.00E-04	2.20E-08
rs883541	A	-0.002	3.00E-04	2.70E-10
rs16942751	A	-0.003	5.00E-04	2.20E-09
rs1719934	A	0.003	3.00E-04	2.60E-17
rs4940525	T	0.003	3.00E-04	4.10E-13
rs2974751	A	0.002	3.00E-04	4.40E-08
rs8101667	T	0.004	3.00E-04	9.30E-44
rs113445505	T	0.004	3.00E-04	7.00E-27
rs281380	T	-0.002	3.00E-04	2.90E-10
rs34647824	A	-0.002	4.00E-04	4.00E-08
rs11123169	T	0.003	3.00E-04	9.20E-15
rs17050272	A	-0.002	3.00E-04	1.10E-12
rs11694902	A	0.004	5.00E-04	1.10E-16
rs7425436	A	0.002	3.00E-04	5.20E-13
rs4664475	T	-0.002	3.00E-04	4.60E-11
rs807624	T	0.003	3.00E-04	7.10E-23
rs35472707	T	-0.007	8.00E-04	6.20E-19
rs187355703	C	0.010	0.0011	1.00E-18
rs35284526	A	0.003	3.00E-04	6.20E-17
rs4666821	T	0.002	3.00E-04	2.50E-11
rs4491726	A	0.003	4.00E-04	5.50E-19
rs60980181	A	-0.003	4.00E-04	1.80E-10

rs1047891	A	-0.007	4.00E-04	1.20E-75
rs1548945	T	0.004	3.00E-04	8.40E-31
rs1050816	T	0.003	3.00E-04	1.10E-15
rs3791221	A	0.002	3.00E-04	1.20E-11
rs13003198	T	0.002	3.00E-04	3.10E-08
rs780093	T	0.004	3.00E-04	1.60E-46
rs2301343	T	-0.002	4.00E-04	4.10E-10
rs2971880	A	-0.002	3.00E-04	7.60E-15
rs10197255	A	0.002	3.00E-04	1.20E-08
rs6546869	A	0.006	4.00E-04	5.10E-48
rs62187537	T	0.004	7.00E-04	9.20E-09
rs1041606	T	-0.002	4.00E-04	2.50E-08
rs2273684	T	0.003	3.00E-04	7.80E-25
rs17216707	T	-0.005	4.00E-04	1.10E-33
rs2235826	A	-0.003	4.00E-04	6.80E-15
rs1407040	T	0.002	3.00E-04	1.40E-08
rs35636653	T	0.002	3.00E-04	2.30E-11
rs72629024	C	0.004	5.00E-04	2.00E-13
rs1509117	A	0.002	4.00E-04	7.10E-10
rs2823139	A	-0.003	3.00E-04	5.20E-16
rs2834317	A	-0.004	5.00E-04	4.30E-14
rs2244237	T	0.003	4.00E-04	6.30E-11
rs131263	T	0.002	4.00E-04	2.20E-11
rs80576	A	-0.003	5.00E-04	1.30E-09
rs4820324	C	-0.002	3.00E-04	5.10E-14
rs112880707	T	0.005	5.00E-04	4.90E-31
rs738527	T	0.003	3.00E-04	4.20E-21
rs2289746	T	-0.002	3.00E-04	2.50E-09
rs9868185	A	0.003	3.00E-04	5.00E-17
rs795009	T	0.002	3.00E-04	7.00E-09
rs10934754	T	0.002	3.00E-04	1.30E-10
rs35320690	T	-0.003	4.00E-04	3.00E-11
rs6778731	T	-0.002	3.00E-04	3.30E-08
rs7624084	T	0.002	3.00E-04	2.00E-08
rs1397764	A	0.004	3.00E-04	2.50E-37
rs76272256	T	0.002	4.00E-04	4.90E-10
rs56065557	C	-0.003	3.00E-04	4.30E-18
rs9823161	A	0.002	4.00E-04	3.30E-09
rs6779998	A	-0.002	3.00E-04	1.60E-08
rs11914389	T	0.003	3.00E-04	3.10E-22
rs7651407	T	0.003	4.00E-04	2.40E-11
rs2581820	A	0.002	3.00E-04	7.90E-10
rs3774726	T	-0.002	3.00E-04	3.70E-11
rs3775932	A	-0.002	3.00E-04	2.00E-09
rs223471	C	0.003	3.00E-04	5.90E-19
rs71606723	A	0.003	4.00E-04	3.40E-12
rs16874073	T	-0.005	7.00E-04	6.60E-11
rs75501914	A	0.004	6.00E-04	9.00E-11
rs4864890	T	-0.002	4.00E-04	2.50E-09
rs12509595	T	-0.004	3.00E-04	6.40E-25
rs12777	C	0.005	9.00E-04	1.10E-08
rs12163971	A	-0.003	4.00E-04	1.70E-12
rs11743174	T	0.002	3.00E-04	1.30E-08
rs3812036	T	-0.007	4.00E-04	2.40E-74
rs13157326	A	-0.003	3.00E-04	1.70E-15
rs1362800	T	-0.005	3.00E-04	5.80E-51
rs12520984	C	0.002	3.00E-04	5.30E-09
rs79760705	T	0.006	5.00E-04	6.50E-25
rs13159523	A	-0.002	3.00E-04	3.20E-13
rs72759880	T	-0.006	5.00E-04	1.10E-26
rs2010352	A	-0.002	3.00E-04	1.70E-09
rs3797537	A	0.002	3.00E-04	2.90E-08

rs1857859	A	0.002	3.00E-04	2.60E-08
rs1268168	A	0.002	3.00E-04	5.40E-14
rs7740107	A	0.003	4.00E-04	8.90E-13
rs9375818	A	-0.003	4.00E-04	6.10E-18
rs3822939	A	-0.003	3.00E-04	2.20E-16
rs9397738	A	0.003	4.00E-04	3.50E-10
rs3765502	T	0.002	4.00E-04	4.00E-08
rs13200335	A	0.002	3.00E-04	8.10E-15
rs77915916	A	0.005	6.00E-04	7.30E-14
rs881858	A	-0.005	3.00E-04	2.90E-55
rs720989	T	0.002	4.00E-04	1.80E-08
rs12212034	T	-0.002	3.00E-04	1.00E-08
rs6458868	T	-0.002	3.00E-04	1.20E-09
rs3925003	T	-0.002	3.00E-04	2.00E-09
rs11755724	A	0.003	4.00E-04	1.10E-13
rs72912510	A	-0.002	4.00E-04	6.40E-09
rs35154268	A	-0.002	4.00E-04	9.90E-09
rs3757387	T	0.003	3.00E-04	7.00E-20
rs62435145	T	-0.006	4.00E-04	2.30E-59
rs62491533	T	-0.003	4.00E-04	1.10E-11
rs10254101	T	-0.007	4.00E-04	1.80E-67
rs12671694	T	0.003	3.00E-04	2.20E-16
rs868822	T	0.003	3.00E-04	2.20E-18
rs6968554	A	-0.002	3.00E-04	1.10E-09
rs3750081	T	-0.002	3.00E-04	1.90E-12
rs700753	C	0.003	3.00E-04	2.10E-20
rs801193	T	-0.002	3.00E-04	1.90E-09
rs41301394	T	0.002	3.00E-04	3.70E-12
rs6973656	A	0.004	3.00E-04	5.70E-28
rs2954017	T	0.002	3.00E-04	1.70E-12
rs34861762	T	-0.004	3.00E-04	4.10E-41
rs10102889	C	-0.004	6.00E-04	6.70E-09
rs1533059	A	0.003	3.00E-04	1.20E-14
rs2976178	C	-0.003	3.00E-04	7.80E-14
rs1321917	C	-0.002	3.00E-04	1.40E-13
rs7024579	T	0.002	4.00E-04	8.20E-11
rs12377027	A	-0.003	5.00E-04	2.90E-08
rs544169	A	0.002	3.00E-04	3.90E-11
rs2039424	A	0.004	3.00E-04	2.10E-44

Four SNPs (rs55929207, rs7169629, rs8096658, and rs10865189) were palindromic SNPs with allele frequency close to 0.5, so they were excluded.
